# The spiritual care intervention “In dialogue with your life story”: Results of a longitudinal study on palliative clients’ spiritual wellbeing

**DOI:** 10.1177/02692163251319143

**Published:** 2025-02-19

**Authors:** Anke I. Liefbroer, Annemarie Foppen, Iris R. Wierstra, Ineke Nagel

**Affiliations:** 1 Department of Religion and Practice, Tilburg School of Catholic Theology, Tilburg University, Utrecht / Tilburg, The Netherlands; 2 Department of Beliefs and Practices, Faculty of Religion and Theology, Vrije Universiteit Amsterdam, Amsterdam, The Netherlands; 3 Chair group Humanist Chaplaincy Studies for a Plural Society, University of Humanistic Studies, Utrecht, The Netherlands; 4 Department of Sociology, Faculty of Social Sciences, Vrije Universiteit Amsterdam, Amsterdam, The Netherlands

**Keywords:** Palliative care, longitudinal studies, spiritual care, spiritual wellbeing, spirituality, chaplains

## Abstract

**Background:**

Spiritual care is important for palliative care, but the evidence base for spiritual care provision is low.

**Aim:**

To investigate the course over time of clients’ spiritual wellbeing who participated in the spiritual care intervention “In dialogue with your life story.”

**Design:**

The intervention consisted of six individual sessions between client and chaplain of various faiths. A longitudinal study was conducted pre- and post-intervention, and a follow-up approximately 10 weeks after post-intervention. Spiritual wellbeing was measured using the EORTC QLQ-SWB32 and NEIS, and symptoms of anxiety and depression as secondary outcome measure using the HADS. Latent growth modeling was used to investigate changes in outcome measures over time.

**Setting/Participants:**

Adult clients receiving home-based, palliative care were eligible to participate in this study.

**Results:**

A total of 75 clients and 33 chaplains participated. On the four EORTC QLQ-SWQ32-subscales, a significant increase was found over time on “relationship with self,” “relationship with others,” and ‘“existential wellbeing” (linear trends). “Relationship with someone or something greater” significantly increased over time but decreased 10 weeks post-intervention (quadratic trend). On the two NEIS-subscales, ego-integrity significantly increased over time (linear trend), while despair significantly decreased (quadratic trend). On the two HADS-subscales, symptoms of anxiety significantly decreased over time (linear trend). No significant change was found for depressive symptoms.

**Conclusions:**

We provided first empirical evidence for an increase in clients’ spiritual wellbeing after enrollment in the spiritual care intervention “In dialogue with your life story.” Future research using control conditions is needed to investigate its causal effect.

**What is already known about the topic?**

• People diagnosed with a life-limiting illness have increased spiritual needs.
• Addressing spiritual needs has been part of the WHO’s palliative care definition for several decades now.
• Despite this recognition, the evidence base for spiritual care provision is low.

**What this paper adds**

• This paper adds to evidence-based spiritual care provision by reporting on palliative clients’ spiritual wellbeing over time before and after enrollment in the spiritual care intervention “In dialogue with your life story.”
• It provides the first empirical evidence for an increase in palliative clients’ spiritual wellbeing after enrollment in the spiritual care intervention.
• The increase in spiritual wellbeing over time continued for at least 10 weeks for four out of six measures of spiritual wellbeing.

**Implications for practice, theory or policy**

• Chaplains may implement this intervention in their practice to alleviate clients’ spiritual needs.
• Future research is necessary to compare the effectiveness of this intervention with other forms of spiritual care or no spiritual care.

## Introduction

Having a serious, life-limiting disease affects not only people’s physical, psychological, and social wellbeing, but also their spiritual wellbeing.^1^ Following the Spiritual Care Task Force of the European Association for Palliative Care (EAPC), we define spirituality as “the dynamic dimension of human life that relates to the way persons (individual and community) experience, express and/or seek meaning, purpose and transcendence, and the way they connect to the moment, to self, to others, to nature, to the significant and/or the sacred.”^2,3^ Studies indicate that as clients’ physical wellbeing deteriorates their spiritual wellbeing may fluctuate over time, and clients often experience heightened spiritual distress at specific moments throughout the trajectory (i.e. at diagnosis, disease progression, terminal stage).^4,5^ Addressing clients’ spiritual needs throughout the illness trajectory has thus been part of the palliative care definition of the World Health Organization for several decades now.^6^

Despite this recognition, the evidence base for spiritual care provision in palliative care is low. Literature review studies into spiritual care in palliative care^7–10^ indicate that, thus far, few randomized controlled trials have been conducted, and their findings are inconclusive. Studies using other designs do show promising results, as they associate spiritual care in palliative care with various positive outcomes for clients, such as the easing of discomfort,^11^ and an increase in spiritual wellbeing and quality of life.^12^ More research is needed to enhance the evidence base of such findings and to show whether spiritual care in palliative care does indeed lead to positive outcomes for clients’ wellbeing over time.

This paper adds to evidence-based spiritual care by reporting on palliative clients’ spiritual wellbeing over time before and after enrollment in the spiritual care intervention “In dialogue with your life story.” In the Netherlands, clients with a life-limiting disease are increasingly required to live at home for as long as possible.^13^ However, as these clients experience spiritual needs beyond the hospital setting,^14^ since 2019 the government has started funding home-based spiritual care for clients in palliative care by chaplains (specialists in the field of spiritual care^3^).^15^ In this home-based context, chaplains encounter clients from various religious or spiritual (R/S) backgrounds, for whom they care for during several weeks, months, or even longer.^16^ The intervention is therefore designed as an interfaith intervention for clients and chaplains from diverse R/S backgrounds and comprises six weekly, 1 h individual sessions at the client’s home.^17^ Key elements are life review, making use of materiality, ritual and embodied experience, and imagination. These are derived from spiritual care tools and interventions designed for other populations, which have been described in previous studies.^18–24^ In this paper, we report on the course of clients’ spiritual wellbeing while participating in this intervention.

## Methods

### Study design

A longitudinal, quantitative design was used to investigate the progression of spiritual wellbeing before and following clients’ enrollment in the spiritual care intervention. A detailed description of the study’s original randomized controlled trial design comparing three groups and ethical considerations was published previously.^25^ Due to COVID-19, the project, which ran from 2019 to 2022, encountered various challenges. These included difficulties in data collection, such as a decline in new applications for chaplaincy care, as clients were hesitant to receive home care during the pandemic. Additionally, there were delays in ongoing trajectories because chaplains were unable to visit clients for an extended period. This situation limited the possibilities to include a sufficient number of participants within the available time. Moreover, randomly recruiting participants and assigning them to one of the three groups turned out to be impossible, as the centers facilitating home-based spiritual care varied significantly in how they assigned clients to chaplains during the pandemic. Therefore, the project shifted from a randomized controlled trial design to a longitudinal design following only the intervention group over time.

### Setting

The study focused on clients receiving home-based, palliative care by chaplains in the Netherlands. In this setting chaplains are self-employed and collaborate in Centers for Questions of Meaning in Life (in Dutch: *Centra voor Levensvragen*). They work from various religious/spiritual (R/S) orientations, for example, as Protestant, Catholic, Muslim, Humanistic, or unaffiliated chaplain, and provide care to clients holding diverse R/S orientations.

### Population

Clients aged 18 years and older and diagnosed with a life-limiting disease were eligible to participate in this study. Exclusion criteria were having a current psychiatric disorder, a life expectancy of less than 6 months, cognitive disability (e.g. a diagnosis of dementia, intellectual disability), insufficient proficiency in the Dutch language, and not being able to speak or interact for longer than 60 min, even with short breaks. These criteria were chosen as they may interfere with participating in the intervention and/or for ethical reasons.

### Sampling

Chaplains were recruited through purposive sampling, as a representative sample was aimed for in terms of their R/S orientation, gender, age, experience as a chaplain, and geographical location. To increase this study’s external validity, as much chaplains as possible could participate. Clients were recruited following convenience sampling. The aim was to include as much clients as possible within the COVID-19 circumstances, but with a minimum sample size of *n* = 53 (see^25^ for a sample size calculation of the original design).

### Recruitment

Chaplains were recruited via coordinators of Centers for Questions of Meaning in Life and participated on a voluntary basis. Eligible clients were asked to participate in this study by their chaplain, home care organization, the coordinator of the Center for Questions of Meaning in Life, or through announcement via client associations. Clients who were interested received a letter at home from the researcher containing information about the study, the informed consent form and the survey (T0). If clients and chaplains weren’t already paired (i.e. because they were recruited via client associations), they were paired based on geographical location. Clients and chaplains gave written informed consent for participation.

### Intervention

The aim of the intervention “In dialogue with your life story” is to improve palliative clients’ spiritual wellbeing.^17^ The intervention consists of six weekly, 1 h individual sessions between client and chaplain. Key elements of the intervention are life review; materiality, ritual and embodied experience; and imagination.^18–24^ These elements are applied in a structured manner, including exercises and homework assignments. The first session focuses on getting to know one another, exploring the clients’ current situation, and explaining the intervention itself. The second, third, and fourth sessions focus on the client’s life story and identity (session #2), relations with others (session #3), and connection with something greater than ourselves (session #4). The themes of hope, despair, gratitude, and regret are central in sessions #5 and #6. See Table 1 for a structured description of the intervention based on the TIDieR checklist.^26^ For a detailed description of the development and theoretical underpinnings of this intervention, see Wierstra et al.^17^ Chaplains received a one-session training to acquaint them with the intervention.

**Table 1.** Description of intervention “In dialogue with your life story” based on the TIDieR checklist.^26^.

Item	Description
Brief name	In dialogue with your life story
Why	The aim of the intervention was to improve palliative clients’ spiritual wellbeing. Key elements of the intervention are life review; materiality, ritual and embodied experience; and imagination. They are derived from spiritual care tools and interventions designed for other populations, which have been described in previous studies.^19–25^ The first session focused on getting to know one another, exploring the clients’ current situation, and explaining the intervention itself. The second, third, and fourth sessions focused on the client’s life story and identity (session #2), relations with others (session #3), and connection with something greater than ourselves (session #4). The themes of hope, despair, gratitude, and regret were central in sessions #5 and #6.
What	The intervention was provided in a structured manner, including exercises and homework assignments for clients. There is a Dutch guide for chaplains (which can be accessed here: https://palliaweb.nl/getmedia/34784b7d-c79c-4f8e-bd0a-335498d88207/In-dialoog-met-je-levensverhaal-Gespreksmodel-geestelijk-verzorgers.pdf) and a workbook for clients (which can be accessed here: https://palliaweb.nl/getmedia/7ed8ef68-5718-4124-b552-01745ba61072/In-dialoog-met-je-levensverhaal-patientenWerkboek-online.pdf).
Who provided	The intervention was developed for chaplains. Although the intervention’s guide provides detailed information on how the intervention is to be performed and can be used as such, chaplains participating in the study followed a 2 h training to acquaint themselves with the intervention, its theoretical background and practical application.
How	The intervention was intended for individual, face-to-face encounters.
Where	The intervention took place at the client’s home.
When and how much	The intervention consisted of six sessions that were planned on a weekly basis. Each session took approximately 1 h.
Tailoring	The intervention included discussions of central themes, homework assignments and exercises during the sessions. The content of these is tailored to the client’s situation.
Modifications	No modifications were made during the course of the study.
How well	After each session, chaplains made notes in a reflection report, including questions about their adherence to the intervention. Also, a selection of participating chaplains was interviewed about their experiences with the intervention.

### Data collection

A quantitative survey was distributed at three moments in time: pre- (T0) and directly post-intervention (T1), and a follow-up (T2) approximately 10 weeks after T1. See Table 2 for a SPIRIT diagram for the schedule of enrollment, intervention, and assessments.^27^ Results on the qualitative data collected as part of the broader project are described elsewhere.^28,29^

**Table 2.** SPIRIT diagram for the schedule of enrollment, intervention, and assessments.

	Study period				
	Enrollment	Baseline	Intervention period	Post-intervention	10 weeks follow-up
Timepoint	T-1	T0		T1	T2
Enrollment:					
Eligibility screen	X				
Informed consent	X				
Allocation		X			
Intervention:					
* In dialogue with your life story*			X		
Assessments:					
* Survey demographic data (clients & chaplains)*		X			
*Survey outcome variables (clients)*: - *Spiritual wellbeing (EORTC & NEIS)* - *Symptoms of anxiety and depression (HADS)*		X		X	X
*Qualitative data: Reflection notes (clients & chaplains)* ^a^			X		
*Qualitative data: Interviews (clients & chaplains)* ^a^				X	

a Results on the qualitative data of this study are described elsewhere.^28,29^

Spiritual wellbeing was the primary outcome. It was measured by using two validated instruments that consist of scales reflecting the central themes of the intervention. The validated Dutch version of the EORTC QLQ-SWB32 was used to measure spiritual wellbeing by focusing on several subscales: “relationship with self” (EORTC_RS), “relationships with others” (EORTC_RO), “relationship with someone or something greater” (EORTC_RSG), and “existential” (EORTC_EX). The instrument consists of 23 scoring items for the scales, and previous studies report good reliability (Cronbach’s alpha ranging from 0.68 to 0.84).^30,31^ Ego-integrity and despair (central in sessions #5 and #6) were measured using the Dutch version of the Northwestern Ego-integrity Scale (NEIS).^32^ These two constructs consisted of four (NEIS_ei) and five items (NEIS_dp). In previous research, the reliability of the scales was sufficient (Cronbach’s alpha 0.72 and 0.61).^32^

As palliative clients are at increased risk of experiencing anxiety and depression,^33,34^ it was hypothesized that the intervention might not only improve clients’ spiritual wellbeing but potentially also clients’ emotional wellbeing. The Dutch version of the Hospital Anxiety and Depression Scale (HADS)^35,36^ was used as a secondary outcome. Symptoms of anxiety (HADS_anx) and depression (HADS_dep) were measured by seven items each, and a previous study reported sufficient reliability for these scales (Cronbach’s alpha for depression ranges from 0.67 to 0.90; Cronbach’s alpha for anxiety ranges from 0.68 to 0.93).^37^

At baseline, demographic data, including clients’ religious/spiritual affiliation and medical data, were collected for descriptive purposes.

### Data analyses

The statistical software program SPSS was used for descriptive analyses. Latent growth modeling (LGM) in MPlus (8, version 1.8.8)^38^ was used to test (linear and non-linear) trends in outcome measures over time, including clustering within chaplains as a random effect to correct the standard errors due to possible similarities/resemblance between clients of the same chaplain. MPlus has the advantage over using Mixed Models in SPSS that clustering can be added to the model. Also, all valid information is used (in case of missing values resulting from (partial) non-response), including that from auxiliary variables. In this study educational level was used as auxiliary variable, since this was the only control variable that significantly related to drop-out. The dataset was organized hierarchically, with observations for each chaplain per client at several moments in time. For each outcome measure, three models were tested and compared to each other: the intercept model (A), the intercept with slope model to investigate a linear trend (B), and the intercept with slope and slope^2^ model to investigate a non-linear (quadratic) trend (C). To estimate models B and C, a design with restrictions was used, with error variances set equal over time and covariances fixed at 0, (only) if the covariance between intercept and slope was not significant. Which of the models best fitted our data was decided based on the fit measurements (RMSEA, CFI, and TLI), *p*-values for slope and slope^2^, and *p*-values for the improvement of the Chi^2^-tests of models A over B and models B over C. As Chi^2^-testing is not possible with clustering, the improvement between Chi^2^-tests was based on the models without clustering.

## Results

### Study population

Data was collected from December 2020 until June 2022. A total of 75 clients participated in this study. Of these, 14 clients (19%) filled out only one of the three surveys, a percentage resembling drop-out rates in other research among palliative clients (20%).^39^ Most clients suffered from COPD or another chronic lung disease (*n* = 22; 30%), cancer (*n* = 21; 29%), or cardiovascular diseases (*n* = 14; 19%). Various other diseases were reported as well, such as kidney failure, ALS, Parkinson’s disease, rheumatism, and MS.

Clients were guided by 33 chaplains. Most of them provided the intervention to one (*n* = 10), two (*n* = 11), or three clients (*n* = 7), and few to four (*n* = 3) or five (*n* = 2) clients. For an overview of participants’ characteristics, see Table 3.

**Table 3.** Overview of participants’ characteristics.

	Patients (*n* = 75)^a^				Chaplains (*n* = 33)^a^		
	*N* (%)	*M*	SD		*N* (%)	*M*	SD
Gender							
Male	18 (25)				6 (21)		
Female	54 (75)				22 (79)		
Age		62.5	12.8			52.9	10.5
Religious/spiritual affiliation							
Not applicable	22 (31)				1 (3)		
Catholicism	14 (20)				3 (10)		
Protestantism	14 (20)				9 (31)		
Islam	1 (1)				1 (3)		
Judaism	1 (1)				0		
Buddhism	2 (3)				2 (7)		
Hinduism	0				0		
Humanism	6 (9)				1 (3)		
Other^b^	3 (4)				2 (7)		
Combination of the above	8 (11)				10 (35)		
Educational level				Endorsement			
Primary school	1 (1)			Not applicable	2 (7)		
Lower vocational	6 (8)			Catholicism	1 (3)		
Lower secondary vocational	6 (8)			Protestantism	11 (38)		
				Buddhism	1 (3)		
Secondary vocational	9 (13)			Humanism	2 (7)		
				RING-GV^c^	12 (41)		
Higher secondary	6 (8)			Years of work experience		13.7	12.7
Higher professional	27 (38)						
University	17 (24)			Number of patients supervised		2.3	1.2

a The numbers do not always add up to resp. *n* = 75/n = 33, due to missing data.

b Such as “universe,” “spirituality,” “animism/’mother” nature’, “mysticism” and “shamanism.”

c RING-GV is a Dutch organization that examines the spiritual competency of chaplains; oftentimes this is done for chaplains who are not authorized or ordained by a religious or humanistic organization.

### Primary outcome: Spiritual wellbeing

Descriptive statistics for the outcome measures at each moment in time are presented in Table 4. For the results of models B and C, see Table 5. For an overview of all statistical analyses, see Supplemental File 1.

**Table 4.** Overview of descriptive statistics for outcome measures.

Outcome measure	Time	Range	*N*	*M*	SD
EORTC_RS	T0	1–4	72	2.86	0.60
	T1	1–4	59	3.07	0.64
	T2	1–4	58	3.11	0.51
EORTC_RO	T0	1–4	72	2.86	0.64
	T1	1–4	59	2.99	0.68
	T2	1–4	58	2.97	0.72
EORTC_RSG	T0	1–4	72	2.34	0.69
	T1	1–4	59	2.46	0.63
	T2	1–4	58	2.39	0.66
EORTC_EX	T0	1–4	72	2.56	0.57
	T1	1–4	59	2.70	0.68
	T2	1–4	58	2.73	0.69
NEIS_ei	T0	0–5	73	3.00	0.94
	T1	0–5	59	3.26	1.02
	T2	0–5	58	3.25	1.10
NEIS_dp	T0	0–5	73	2.10	1.02
	T1	0–5	60	1.70	1.06
	T2	0–5	58	1.88	1.10
HADS_anx	T0	0–3	73	1.07	0.59
	T1	0–3	60	0.91	0.63
	T2	0–3	58	0.81	0.56
HADS_dep	T0	0–3	73	0.91	0.57
	T1	0–3	60	0.79	0.66
	T2	0–3	58	0.77	0.56

**Table 5.** Overview of outcome measures over time based on model B (linear) and C (quadratic; *n* = 75).

Variable	Model^a^	RMSEA	CFI	TLI	Intercept	SE	Slope	SE	*p*	Slope2	SE	*p*
EORTC_RS	**B**	**0.009**	**0.999**	**0.999**	**2.883**	**0.064**	**0.103**	**0.046**	0.026*	**n.a.**	**n.a.**	**n.a.**
	C	0.000	1.000	1.000	2.866	0.068	0.227	0.145	0.117	−0.062	0.068	0.362
EORTC_RO	**B**	**0.050**	**0.990**	**0.993**	**2.882**	**0.070**	**0.077**	**0.033**	0.021*	**n.a.**	**n.a.**	**n.a.**
	C	0.000	1.000	1.000	2.859	0.075	0.252	0.125	0.043*	−0.089	0.056	0.115
EORTC_RSG	B	0.197	0.946	0.959	2.382	0.098	0.035	0.028	0.207	n.a.	n.a.	n.a.
	**C**	**0.099**	**0.993**	**0.990**	**2.347**	**0.097**	**0.314**	**0.089**	< 0.001**	**−0.141**	**0.041**	0.001**
EORTC_EX	**B**	**0.115**	**0.927**	**0.945**	**2.578**	**0.064**	**0.083**	**0.041**	0.044*	**n.a.**	**n.a.**	**n.a.**
	C	0.101	0.972	0.957	2.560	0.074	0.227	0.173	0.189	−0.073	0.079	0.355
NEIS_ei	**B**	**0.000**	**1.000**	**1.000**	**3.034**	**0.088**	**0.142**	**0.058**	0.013*	**n.a.**	**n.a.**	**n.a.**
	C	0.000	1.000	1.000	3.000	0.093	0.400	0.182	0.028*	−0.131	0.097	0.180
NEIS_dp	B	0.162	0.785	0.839	2.042	0.113	−0.106	0.060	0.077	n.a.	n.a.	n.a.
	**C**	**0.000**	**1.000**	**1.000**	**2.119**	**0.125**	**−0.689**	**0.256**	0.007**	**0.295**	**0.118**	0.013*
HADS_anx	**B**	**0.147**	**0.982**	**0.986**	**1.066**	**0.063**	**−0.139**	**0.040**	< 0.001**	**n.a.**	**n.a.**	**n.a.**
	C	0.000	1.000	1.000	1.074	0.064	−0.125	0.133	0.107	0.041	0.060	0.491
HADS_dep	B	0.204	0.935	0.951	0.902	0.066	−0.074	0.039	0.058	n.a.	n.a.	n.a.
	C	0.000	1.000	1.000	0.912	0.068	−0.178	0.149	0.232	0.057	0.064	0.377

a Model B = intercept + slope (linear). Model C = intercept + slope + slope^2^ (quadratic). Bold text indicates the model that best fitted our data for that outcome measure.

* Significant at *p* < 0.05.

** Significant at *p* < 0.01.

**Figure 1.**
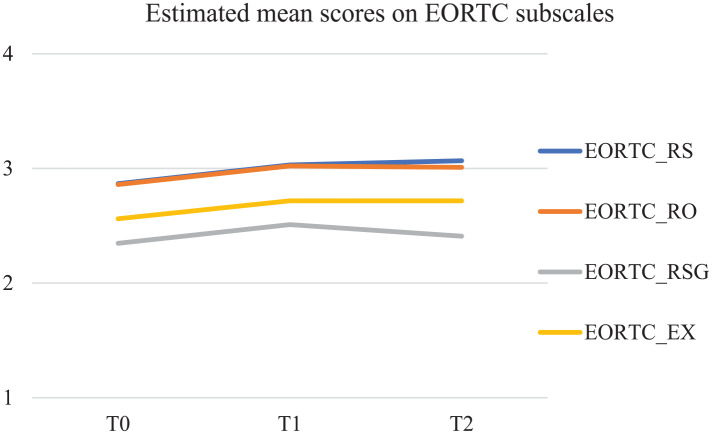
Estimated mean scores in MPlus on EORTC QLQ-SWQ32 subscales.

Results of the latent growth modeling (LGM) show significant increases in spiritual wellbeing over time. On the subscales of the EORTC QLQ-SWB32 (see Figure 1), relationship with self (EORTC_RS), relationships with others (EORTC_RO), and existential wellbeing (EORTC _EX) significantly increased over time with a linear trend (resp. *S* = 0.103; *p* = 0.026; *S* = 0.077; *p* = 0.021; *S* = 0.083; *p* = 0.044). The relationship with someone or something greater (EORTC_RSG) significantly increased over time as well, but with a decline after T1 (*S* = 0.314; *p* < 0.001; S^2^ = −0.141; *p* = 0.001).

**Figure 2.**
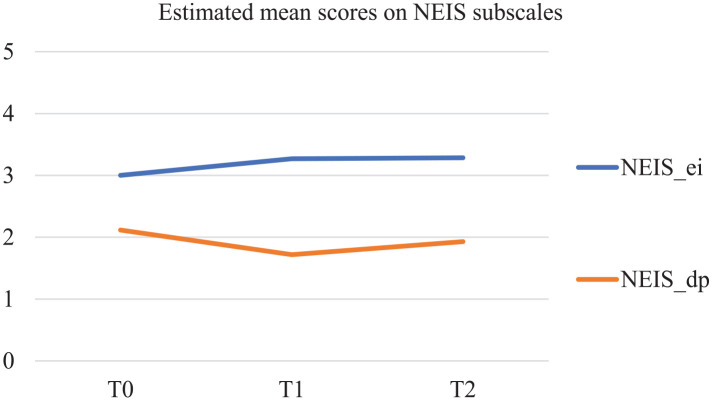
Estimated mean scores in MPlus on NEIS subscales.

The two subscales of the NEIS also show significant developments over time (see Figure 2). Ego-integrity (NEIS_ei) significantly increased over time following a linear trend (*S* = 0.142; *p* = 0.013), and despair (NEIS_dp) significantly decreased over time, but, following a quadratic trend, with a slight rise again after T1 (*S* = −0.689; *p* = 0.007; *S*^2^ = 0.295; *p* = 0.013).

### Secondary outcomes: Symptoms of anxiety and depression

**Figure 3.**
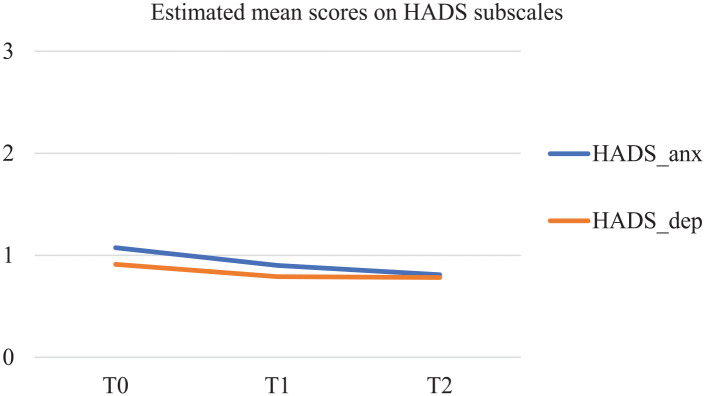
Estimated mean scores in MPlus on HADS subscales.

Results of the latent growth modeling (LGM) on the HADS (see Figure 3) indicate a significant decrease in symptoms of anxiety over time following a linear trend (HADS_anx; *S* = −0.139; *p* < 0.001). No significant trend was found over time for depressive symptoms.

## Discussion

This study’s aim was to examine the course of clients’ spiritual wellbeing over time who were enrolled in the spiritual care intervention “In dialogue with your life story.” Based on a longitudinal quantitative survey among 75 clients, the findings show a significant increase in spiritual wellbeing over time. After participating in the intervention, clients report a better relationship with themselves, others, and someone or something greater, and an increase in existential wellbeing, as measured with the EORTC QLQ-SWB32. Ego-integrity, as measured by the NEIS, also significantly increases over time, while feelings of despair decrease. Notably, most of these outcomes show a lasting effect 10 weeks after the intervention took place (linear trend). This is an important finding, as it indicates the relevance of this intervention not only in the short term but also long(er) term. This contrasts with earlier studies in which researchers did not find lasting effects of narrative spiritual interventions on clients’ spiritual wellbeing.^40–42^ One reason for our intervention to show lasting effects on spiritual wellbeing may be the long duration (six sessions) of this intervention, which aligns with the outcomes on spiritual wellbeing in other longer narrative interventions (i.e. six^43^ or eight sessions^44^). A second reason may be found in the characteristics of this intervention itself, which was not limited to narrativity, but included various ways to enhance client’s meaning in life and improve their spiritual wellbeing, specifically by using materiality, ritual and embodied experience, and imagination.^17^

Meanwhile, some outcome measures do suggest trends of stabilization or a decline in the longer term, with two of these deviating significantly from linearity. First, the relationship with someone or something greater significantly increased over time, but this change diminished 10 weeks after the intervention took place, as indicated by its quadratic trend. Chaplains are often described as embodying roles of a companion (emphasizing a relational dimension), a counselor (emphasizing a therapeutic dimension), and a spiritual guide (emphasizing a spiritual dimension).^45–47^ An explanation could, therefore, be that the relationship with someone or something greater is embodied during the intervention by chaplains themselves (in their role as spiritual guide) and that this diminishes once the intervention has ended. Another explanation is found in the content of the intervention—which included a conversation on connectedness with something greater than ourselves (i.e. in session #4)—and in the fact that taking part in the study and intervention itself connects people with something beyond themselves. Both of these may have contributed to heightened feelings of relationality to someone or something greater while participating in the intervention and directly thereafter, but not to lasting effects.

Secondly, the significant decrease in feelings of despair after the intervention did not persist and increased in the 10 weeks post-intervention (quadratic trend). This seems remarkable, given that we did find lasting (linear) effects 10 weeks post-intervention on ego-integrity as well as on other measures of spiritual wellbeing as described above. Another study using a longer-term (eight-sessions) narrative intervention did find lasting effects on both ego-integrity and despair.^44^ Yet, in contrast to our study population which included clients with a life-limiting disease, this other study focused on cancer survivors who had completed their main treatment 6 months prior to the study. An explanation may, therefore, be found in the nature of the palliative phase itself, in which, as the disease progresses, fewer options are available to restore past regrets. Future research is needed to better understand the lasting impact of the intervention on clients’ spiritual wellbeing and, specifically, the effect on feelings of despair.

In addition to changes in clients’ spiritual wellbeing over time, we found a significant decrease over time in clients’ feelings of anxiety, as measured by the HADS (linear trend). This aligns with our hypothesis that as clients’ spiritual wellbeing improves, so does their emotional wellbeing. Yet no such change was identified for feelings of depression. As scores on this measure were already quite low before the intervention took place (*M* < 1, on a scale from 0 to 3), such feelings may not have been present that much among these clients, potentially explaining the lack of decrease in these. Additionally, this finding may be interpreted as an indication that the intervention targeted those clients it intended to target: those receiving palliative care while primarily struggling with existential or spiritual questions (requiring chaplaincy care) rather than symptoms of depression (requiring psychological and/or psychiatric care). Meanwhile, the lack of change in feelings of depression should be interpreted with caution, as the decrease was close to significant (*p* = 0.058). A (slightly) larger sample size may have led to different results here.

### Strengths and limitations

This study has several strengths and limitations regarding its methodological design and the population targeted by the intervention. There is no consensus in the chaplaincy literature on which outcome measures best capture the goals and effects of chaplaincy care.^48–50^ We have chosen to focus on spiritual wellbeing as the concept we are primarily interested in and used two standardized instruments for measuring this (the EORTC QLQ-SWB32 and NEIS). As we found change over time on these outcome measures, our study indicates that these instruments are sensitive to change that occurred in clients’ spiritual wellbeing—which is a methodological strength of this study. However, participating in the intervention may have correlated with other changes in clients’ wellbeing that we have not been able to measure with these instruments. For instance, although the EORTC QLQ-SWB32 and NEIS-subscales measured aspects relating to themes that were addressed in the intervention (e.g. relationship with self and others), the impact of other characteristics of the intervention, such as life review, materiality, or imagination, could not be established by these instruments. Future studies could benefit from the development of outcome measures that more specifically capture the effects of chaplaincy interventions.^41^

A methodological limitation is that we were unable to compare the results with those of a control group. We cannot rule out the possibility that changes in spiritual wellbeing are due to external developments that happened in the research period (like COVID-19-related measures) or to maturation (autonomous developments with age or with the progression of a serious disease).^51^ We encourage researchers to make use of a control group in future studies. Analysis of qualitative data collected as part of a larger project concerning this intervention does indicate that both clients and chaplains ascribe the positive impact of the intervention on clients’ spiritual wellbeing to specific elements of the intervention. These include the drawing of a lifeline, meditating exercises and other work forms, a closing ritual, and the content and structure of the intervention.^29^

The intervention “In dialogue with your life story” is designed as an interfaith intervention—that is, it is intended to benefit clients and chaplains with various religious/spiritual (R/S) orientations.^17^ The diversity of R/S orientations, including non-religious ones, among participants suggests that the intervention indeed targets the spiritual needs of an interfaith population, which is a strength of this study. Nonetheless, the study sample is limited, and therefore possibly biased, in terms of educational level. Clients who participated in this study had a relatively high educational level, and the higher clients’ educational level the less likely they were to drop out during the course of this study. This aligns with other studies indicating a positive correlation between educational level and valuing chaplaincy care.^52,53^ Chaplaincy interventions are urgently needed that are more sensitive to spiritual needs of clients who have a lower educational level. Finally, the study took place in home-based chaplaincy care, whereas spiritual care as part of palliative care is provided in many other settings and by other staff members as well. As the content and characteristics of the intervention are not limited to home-based chaplaincy care, we encourage researchers to study the effects of this intervention in other healthcare settings and to investigate to what extent this intervention can be provided by other staff as well.

## Conclusion

This study provided the first empirical evidence for an increase in clients’ spiritual wellbeing after enrollment in the spiritual care intervention “In dialogue with your life story.” Future research using control conditions is needed to investigate its causal effect.

## Supplemental Material

sj-xlsx-1-pmj-10.1177_02692163251319143 – Supplemental material for The spiritual care intervention “In dialogue with your life story”: Results of a longitudinal study on palliative clients’ spiritual wellbeing Supplemental material, sj-xlsx-1-pmj-10.1177_02692163251319143 for The spiritual care intervention “In dialogue with your life story”: Results of a longitudinal study on palliative clients’ spiritual wellbeing by Anke I. Liefbroer, Annemarie Foppen, Iris R. Wierstra and Ineke Nagel in Palliative Medicine
